# Intelligent Intrusion Detection System Against Various Attacks Based on a Hybrid Deep Learning Algorithm

**DOI:** 10.3390/s25020580

**Published:** 2025-01-20

**Authors:** Bambang Susilo, Abdul Muis, Riri Fitri Sari

**Affiliations:** Department of Electrical Engineering, Faculty of Engineering, Universitas Indonesia, Depok 16424, Indonesia; bambang.susilo91@ui.ac.id (B.S.); muis@ui.ac.id (A.M.)

**Keywords:** deep learning, intrusion detection system, Internet of Things (IoT), autoencoder, long short-term memory (LSTM), convolutional neural network (CNN)

## Abstract

The Internet of Things (IoT) has emerged as a crucial element in everyday life. The IoT environment is currently facing significant security concerns due to the numerous problems related to its architecture and supporting technology. In order to guarantee the complete security of the IoT, it is important to deal with these challenges. This study centers on employing deep learning methodologies to detect attacks. In general, this research aims to improve the performance of existing deep learning models. To mitigate data imbalances and enhance learning outcomes, the synthetic minority over-sampling technique (SMOTE) is employed. Our approach contributes to a multistage feature extraction process where autoencoders (AEs) are used initially to extract robust features from unstructured data on the model architecture’s left side. Following this, long short-term memory (LSTM) networks on the right analyze these features to recognize temporal patterns indicative of abnormal behavior. The extracted and temporally refined features are inputted into convolutional neural networks (CNNs) for final classification. This structured arrangement harnesses the distinct capabilities of each model to process and classify IoT security data effectively. Our framework is specifically designed to address various attacks, including denial of service (DoS) and Mirai attacks, which are particularly harmful to IoT systems. Unlike conventional intrusion detection systems (IDSs) that may employ a singular model or simple feature extraction methods, our multistage approach provides more comprehensive analysis and utilization of data, enhancing detection capabilities and accuracy in identifying complex cyber threats in IoT environments. This research highlights the potential benefits that can be gained by applying deep learning methods to improve the effectiveness of IDSs in IoT security. The results obtained indicate a potential improvement for enhancing security measures and mitigating emerging threats.

## 1. Introduction

### 1.1. Research Background

New developments in communication technology, particularly in the Internet of Things (IoT), have significantly outpaced conventional technologies. Implementing IoT technology can enhance quality of life through modernization and enable collecting, analyzing, and comprehending environmental data. The IoT is a rapidly expanding computing discipline. By the end of 2030, it is predicted that there will be approximately 29 billion IoT devices [1], making IoT one of the most rapidly expanding areas in the computing era.

Intelligent and practical applications, such as intelligent healthcare, home automation, intelligent transport, and intelligent classrooms, are all improved by IoT technology. Simultaneously, due to the wide-ranging and multicomponent nature of IoT systems, new security challenges have arisen in their deployment [2].

Due to their intricate and interdependent nature, ensuring security measures over the vast attack surfaces of IoT systems is a formidable task. Comprehensively addressing security requirements is imperative for finding solutions to these challenges. However, most IoT devices operate without human supervision, rendering them vulnerable to physical access from intruders. These devices are linked via wireless networks; unauthorized individuals can intercept the communication channels and acquire confidential data. The inability of IoT devices to accommodate intricate security structures is due to their constrained computational capabilities and power.

IoT systems need more security due to the exchange of information, their energy sources, and the need for trustworthy interactions within the physical dimension. IoT systems are components of a computer’s physical structure, meaning their operation in a real-world environment might be unexpected and cause disturbances. Independently, IoT systems need to constantly adapt and function predictably, with safety being the primary concern, particularly in settings like healthcare systems, where threatening conditions may arise. Furthermore, the interconnected nature of IoT systems creates new attack surfaces that render them more vulnerable to security breaches than other computing systems. Consequently, traditional security solutions may not be effective in such scenarios. In the past, major cyberattacks, such as distributed denial of service (DDoS) attacks, have affected IoT networks, resulting in significant losses [3]. As stated in [1], the predicted number of IoT devices could reach 29 billion by 2030. A DDoS attack involves using several host hackers to flood the target server with traffic, resulting in complete system failure and denial of service (DoS) for legitimate users attempting to access services on the server.

### 1.2. Overview of Existing Methods

The increasing complexity of the Internet of Things (IoT) and the growing sophistication of cyberattacks have prompted significant research into the application of deep learning techniques in intrusion detection systems (IDSs) for network security. This section provides a detailed overview of existing studies on IoT security challenges, advancements in deep learning, and the development and application of deep-learning-based IDSs in network environments.

The IoT represents a vast network of interconnected devices, including physical and digital objects, individuals, animals, and machines, all transferring data autonomously without human intervention. As IoT networks expand, the volume of data exchanged between devices grows exponentially, creating new security, efficiency, and privacy challenges. One of the critical tasks in maintaining IoT infrastructure is detecting and mitigating potential security breaches, making intrusion detection a cornerstone of effective network management. Traditional IDSs often fall short when faced with the immense scale of IoT-generated data and the increasingly sophisticated nature of cyberattacks. In contrast, recent advancements in deep learning have shown significant potential to address these challenges, offering scalable and adaptive solutions for securing IoT systems.

Despite advancements in intrusion detection systems (IDSs) for IoT networks, existing methods often fail to fully address critical challenges, such as class imbalance, temporal feature extraction, and the integration of static and dynamic data patterns. Conventional approaches typically employ standalone models, which lack the capability to simultaneously process spatial and temporal dimensions of network traffic, leading to suboptimal detection accuracy, particularly for complex or emerging attack types. This study aims to bridge these gaps through a hybrid deep learning framework (AE-LSTM-CNN) that integrates autoencoders (AEs) for static feature extraction, long short-term memory (LSTM) networks for temporal dynamics, and convolutional neural networks (CNNs) for spatial pattern refinement. By leveraging this multistage approach, the proposed model ensures comprehensive feature representation, enabling precise classification of IoT network traffic and enhanced detection of diverse attack types.

#### 1.2.1. Deep Learning

Deep learning has emerged as a powerful tool for managing the large volumes of data generated by IoT systems. Its ability to automatically extract complex data representations makes it an ideal choice for handling the complexities of IoT data [4]. The deep learning methodology also has the potential to facilitate deep linking within IoT ecosystems [5]. Unlike traditional machine learning techniques, which rely on manual feature engineering, deep learning models can analyze data at multiple levels of abstraction, uncovering hidden patterns and relationships [6]. These models employ numerous layers of nonlinear processing units to extract both specialized and generative features, drawing inspiration from neural functions and signal processing principles.

Deep learning methods are broadly categorized into supervised (discriminative), unsupervised (generative), and hybrid approaches. Discriminative techniques, such as recurrent neural networks (RNNs) and convolutional neural networks (CNNs), are commonly used for tasks like classification and prediction. Generative techniques, including restricted Boltzmann machines (RBMs) and deep autoencoders (DAEs), model complex probability distributions and enable the generation of new data samples. Hybrid methods, such as ensembles of deep learning networks (EDLNs) and generative adversarial networks (GANs), combine the strengths of discriminative and generative approaches to enhance overall performance.

CNNs reduce the complexity of traditional neural networks by employing sparse interactions and parameter sharing and maintaining equivariance to transformations. These techniques optimize the model’s performance, although they may introduce challenges during training and scalability [2]. On the other hand, autoencoders (AEs) excel at unsupervised learning by encoding input data into concise representations, which are then reconstructed by a decoder. This process is highly effective for feature extraction, as it captures the most relevant information in the data.

Complementing AEs, long short-term memory (LSTM) networks, a variant of RNNs, are adept at retaining information over extended sequences [3]. LSTMs use gating mechanisms to selectively preserve or discard information, making them particularly effective for analyzing time-series data and sequences. In cybersecurity, LSTM networks utilize dropout and fully connected (FC) layers, paired with activation functions like the sigmoid, to distinguish between normal and abnormal patterns.

Feature selection and extraction are critical in optimizing deep learning models for intrusion detection. While feature selection reduces the number of variables by considering each independently, feature extraction combines and transforms raw features into a condensed set that retains the most significant information. This process enhances the model’s efficiency by reducing computational overhead while preserving essential data [7].

Our approach integrates the strengths of AEs, LSTM networks, and CNNs to address the diverse requirements of data processing in IoT environments. AEs capture static data attributes, LSTMs incorporate temporal dynamics, and CNNs excel at hierarchical feature extraction for classification. Together, these models form a robust framework for efficient data handling, feature engineering, and classification, enabling the proposed system to achieve superior performance in detecting IoT-based intrusions.

#### 1.2.2. IDS

An intrusion detection system (IDS) is a vital technique used to identify potentially malicious actions within a system or network. IDSs are indispensable in monitoring and analyzing network traffic to detect unauthorized access, anomalous behavior, and potential security threats. They analyze both incoming and outgoing traffic, perform various analyses, and identify evidence of attempted intrusions. This capability makes IDSs critical for maintaining system security, especially in increasingly complex network environments.

In IoT environments, the landscape of cyber threats is highly diverse and continuously evolving. Threats, such as brute force attacks, DDoS attacks, DoS attacks, Mirai attacks, recon attacks, spoofing attacks, and web attacks, challenge system security by exploiting different vulnerabilities. The following bullets outline these types of attacks, which are all included in the CICIoT2023 dataset used in this research:

- Brute Force Attacks: Adversaries use exhaustive methods to decipher valid usernames and passwords by systematically trying all possible combinations. Automated tools are often employed to exploit systems with insecure or guessable credentials.
- DDoS (Distributed Denial of Service) Attacks: Multiple compromised systems flood a network with traffic from various sources, overwhelming servers and disrupting legitimate user access.
- DoS (Denial of Service) Attacks: These attacks, similar to DDoS attacks but originating from a single source, disrupt services by overwhelming resources.
- Mirai Attacks: A notable DDoS attack that uses IoT devices as a botnet to launch large-scale attacks, highlighting the vulnerabilities in IoT systems.
- Reconnaissance (Recon) Attacks: Focused on gathering intelligence, these attacks survey systems to identify exploitable vulnerabilities.
- Spoofing Attacks: Attackers falsify IP addresses or identities to deceive systems, imitating legitimate sources to gain unauthorized access.
- Web Attacks: These attacks target web applications and infrastructure, exploiting vulnerabilities to breach and manipulate digital platforms.

Each type of attack poses unique challenges for IoT environments. For example, brute force attacks exploit weak credentials, while DDoS and DoS attacks disrupt services by overwhelming network resources. The Mirai attack in 2016 is a significant example of a DDoS attack, where compromised IoT devices formed a botnet to launch a massive assault, emphasizing the need for stronger IoT security measures.

Reconnaissance attacks further compound the challenge by allowing adversaries to gather intelligence on systems to plan more destructive attacks. Spoofing attacks complement recon attacks by deceiving systems with falsified identities, enabling unauthorized access. Finally, web attacks threaten the application layer by exploiting vulnerabilities to manipulate or compromise the digital infrastructure.

Given this diverse threat landscape, there is a critical need to develop more sophisticated and dynamic defenses. Emerging technologies, particularly deep learning, provide promising solutions by enabling intrusion detection systems to identify patterns, predict potential attacks, and respond adaptively. Such advances are crucial for protecting IoT environments against the increasingly intricate nature of cyber threats.

#### 1.2.3. Research Related to Deep Learning and IDSs on Networks

The integration of deep learning into cybersecurity, particularly in intrusion detection systems (IDSs), has been the recent focus of significant research efforts. These studies demonstrate the effectiveness of deep learning techniques in improving IDS performance by addressing sophisticated attack patterns and network anomalies.

Soe et al. [8] proposed a sequential detection framework for botnet attack recognition using the N-BaIoT dataset to capture snapshots of network activity. This approach included an autoencoder (AE) for feature selection and demonstrated an overall detection accuracy exceeding 99%, highlighting the potential of thin, high-performance detection systems for identifying abnormal traffic originating from compromised devices.

Manumurugan et al. [9] introduced a system architecture based on a deep belief network (DBN) for intrusion detection. Using the CICIDS 2017 dataset, their work targeted attacks like PortScan, DoS/DDoS, brute force, and web attacks. The study showcased the capability of DBN to handle diverse attack types effectively, addressing system vulnerabilities.

Alotaibi et al. [10] employed a stacking deep learning approach using pre-trained ResNet models to identify malicious IoT data traffic. By leveraging datasets like N-BaIoT and ICS, the study demonstrated how fused deep learning models enhance intrusion detection in IoT environments.

Al-Haija et al. [11] presented IoT-IDCS-CNN, a parallel-processing system for IoT network security. The architecture used CNNs and high-performance computing powered by NVIDIA GPUs and an Intel I9-core CPU. Through traffic classification and feature engineering, the system achieved reliable attack detection using the NSL-KDD dataset, which includes common IoT intrusions.

Hiromoto et al. [12] introduced a deep-learning-based architecture incorporating generative adversarial networks (GANs) to distinguish between normal and abnormal system activities. This method showed promising results in detecting anomalous IoT network behavior while emphasizing GANs’ potential in cybersecurity.

Kaur et al. [13] developed D-Sign, a deep learning technique for hybrid threat detection and signature generation. Using NSL-KDD and CICIDS-2017 datasets, D-Sign demonstrated high sensitivity and precision in generating threat signatures for a wide range of attacks.

Other researchers have also explored various deep learning methods to enhance IDSs. For instance, Vishwakarma et al. [14] discussed DoS attack techniques and mitigation mechanisms for IoT networks. Xia et al. [15] proposed a reinforcement learning approach to mitigate DDoS attacks using throttling mechanisms, while Razib et al. [16] developed the DNNLSTM model to defend IoT communications against diverse cyber threats, achieving high performance based on accuracy, the F1 score, precision, and recall. Meanwhile, Liu et al. [17] presented NetSentry, a network intrusion detection system utilizing bidirectional asymmetric LSTM (Bi-ALSTM) to address time-sensitive network threats. Similarly, Pektas et al. [18] combined RNNs and CNNs for botnet localization using ISOT and CTU-13 datasets.

Restuccia et al. [19] provided a comprehensive survey and taxonomy of IoT security research, emphasizing machine learning and software-defined networking applications. Their work highlights the current challenges and future directions in securing IoT systems. Meanwhile, Lopes et al. [20] developed a hybrid IDS combining supervised and unsupervised techniques. Using a denoising autoencoder (DAE) and a DNN classifier, their approach achieved high detection accuracy with the CICIDS2018 dataset.

Gamage et al. [21] evaluated multiple deep learning models, including AE, DBN, feedforward neural networks, and LSTM, for attack classification. Their results showed that feedforward neural networks excelled in the F1 score, accuracy, and inference time when applied to contemporary datasets, such as CIC-IDS2018 and CIC-IDS2017.

Innovative methodologies have also been introduced to tackle the resource constraints of IoT environments. Wang et al. [22] discussed the implementation of an innovative IDS specifically designed for IoT devices. This system, which integrates deep learning techniques with a dynamic quantization process, addresses the limitations posed by the resource constraints of IoT environments. The authors employed a model that combines DNNs with bidirectional long short-term memory (BiLSTM) networks, enhancing its capability to identify and analyze complex attack patterns effectively. This method maintains high detection accuracy, demonstrating superior performance compared to traditional models on benchmark datasets, such as CIC IDS2017, N-BaIoT, and CICIoT2023. By introducing incremental principal component analysis for feature reduction and dynamic quantization to optimize the model, Wang et al. [23] effectively streamlined the intrusion detection process.

Lastly, Khan et al. [23] investigated the application of IDSs in healthcare IoT, utilizing models like random forest, adaptive boosting, and deep neural networks. Their study addressed both binary and multiclass classification tasks, emphasizing the critical need for robust data security in healthcare systems.

### 1.3. Gaps in Existing Methods

The development of effective intrusion detection systems (IDSs) in IoT environments has been a focus of research, with various machine learning and deep learning models proposed to address the complexities of evolving cyber threats. While these models have shown promise, they often excel in isolated aspects, such as spatial or temporal feature extraction, but they lack a comprehensive approach to capturing network traffic’s diverse and dynamic nature. This subsection provides a comparative analysis of the proposed AE-LSTM + CNN framework against state-of-the-art approaches, highlighting its performance advantages, architectural innovations, and contributions to addressing existing gaps in the literature.

- Comparative Performance of the AE-LSTM-CNN Model: The proposed AE-LSTM-CNN model achieves significant improvements in accuracy, precision, recall, and the F1 score compared to existing methods in intrusion detection systems (IDSs). For instance, when evaluated on the CIC IoT 2023 dataset, the model outperforms approaches like the IoT-IDCS-CNN by Al-Haija et al. [11], which relies solely on convolutional neural networks, and the DNN-BiLSTM by Wang et al. [22], which integrates bidirectional LSTM with dense neural networks. Specifically, our model achieves an F1 score of 99.19% compared to 97.00% for IoT-IDCS-CNN and 91.73% for DNN-BiLSTM. This superior performance highlights the ability of the hybrid architecture to effectively capture both temporal and spatial features, providing a more comprehensive analysis of IoT network traffic.
- Advances in Feature Engineering and Attack Detection: In comparison to prior work, the multistage feature extraction in the AE-LSTM-CNN architecture provides a notable advancement in capturing diverse data characteristics. Methods like GAN-based anomaly detection by Hiromoto et al. [12] and autoencoder-based feature selection by Gamage et al. [21] demonstrate strong detection capabilities but are often limited by their focus on static or singular feature representations. Our approach combines the strengths of autoencoders, LSTMs, and CNNs, enabling the model to handle a wider range of attacks, including Mirai and Reconnaissance, with higher precision. This is evident from the detailed performance analysis presented in the result section, where our model shows consistent superiority across attack classes.
- Bridging Gaps in the Prior Literature: The literature has highlighted a need for models capable of addressing imbalanced datasets and combining static and temporal features effectively. The AE-LSTM-CNN model bridges these gaps by introducing a three-stage architecture tailored to the unique characteristics of IoT traffic. While earlier approaches, such as Pektas et al.’s CNN-RNN and Lopes et al.’s DAE-DNN, excel in either temporal or spatial domains, they fail to integrate these capabilities seamlessly. By addressing these limitations, our model not only enhances detection accuracy but also sets a new benchmark for feature integration and attack classification in IoT environments.

These studies collectively illustrate the growing importance of deep learning in enhancing IDS capabilities. Researchers have demonstrated significant improvements in detecting and mitigating complex cybersecurity threats by leveraging advanced architectures, feature selection, and optimization techniques. The following section will detail the specific methodology and approaches employed in our research.

### 1.4. Proposed Methodology and Innovations

The objective of this study is to enhance the performance of current deep learning techniques to detect attacks in IoT environments. Deep learning methods are practical tools for analyzing information within IoT environments, allowing for the study of “normal” and “abnormal” behavior by IoT devices and components as they interact with each other. It is possible to gather and analyze data from every IoT system component to identify typical interaction patterns, enabling the early detection of malicious behavior. Furthermore, machine learning/deep learning techniques are valuable in anticipating new attacks that may be variations of previous ones because they can predict upcoming attacks by studying historical samples. This paper presents a comprehensive evaluation of attacks using the CIC-IoT 2023 dataset. It introduces a novel framework that integrates three complementary neural network architectures: autoencoders (AEs), long short-term memory (LSTM) networks, and convolutional neural networks (CNNs). The proposed integration strategically leverages the unique strengths of each architecture to perform robust feature engineering and precise classification. First, AEs are employed to generate compressed representations of raw data, a process critical for efficient data processing and analysis. By encoding input data into concise formats, AEs excel at extracting unsupervised features from both benign and attack datasets, setting the foundation for subsequent stages of analysis.

Building upon these compressed representations, LSTM networks enrich the features by capturing long-term dependencies and temporal dynamics within the data. This capability is especially vital for IoT scenarios where sequence prediction and time-based patterns play a significant role in detecting malicious behavior. LSTMs’ effectiveness in processing sequential data makes them indispensable for applications involving time-series data, video, text, and other temporally dependent domains.

The enriched features from AEs and LSTMs are then utilized by CNNs, which further enhance the feature extraction process. Known for their ability to process structured, grid-like data, such as images and text, CNNs are adept at identifying complex patterns and distinguishing between benign and attack data. By integrating these refined features, CNNs simplify the classification task, effectively differentiating between various input types.

This strategic combination of AEs, LSTMs, and CNNs forms a cohesive and comprehensive analytical framework. AEs and LSTMs collaborate to extract and refine features, while CNNs capitalize on these enriched features for accurate classification. However, the approach comes with computational demands and requires tailoring to the specific characteristics of the dataset to ensure optimal performance. This research highlights the potential of combining specialized neural network architectures to address the unique challenges of IoT attack detection, offering a powerful yet adaptable solution for securing IoT environments.

Combining an AE, LSTM, and CNN within a hybrid architecture has demonstrated superior performance compared to other approaches, as measured by recall, accuracy, the F1 score, and precision. Our findings highlight that this integration leads to greater detection accuracy and improved performance metrics, reinforcing the effectiveness of the proposed approach.

The AE-LSTM-CNN framework is specifically designed to address the limitations of existing IDS approaches by introducing a multistage architecture that combines the strengths of three complementary neural network models. Autoencoders (AEs) are utilized to reduce data dimensionality while retaining essential static features, which is particularly critical for handling noisy datasets like CIC IoT 2023. This step ensures that the framework captures intrinsic characteristics of the data that might be overlooked by traditional models.

Building on the output of AEs, LSTM networks capture temporal dependencies within the data, enabling the detection of time-series patterns often indicative of cyberattacks. This temporal modeling is crucial in IoT networks, where sequences of events, rather than isolated anomalies, typically signify malicious activity. Finally, CNNs refine and enhance these extracted features by identifying intricate spatial relationships, ensuring that the final classification is both robust and accurate.

By integrating these components, the proposed framework overcomes the limitations of standalone models, such as CNNs’ inability to capture sequential dependencies or LSTMs’ focus on temporal data without accounting for spatial patterns. This holistic approach ensures that the system can adapt to the dynamic and heterogeneous nature of IoT environments, providing higher detection accuracy and generalizability across diverse attack types.

This study makes the following key contributions to the literature:

1. Advancement in Feature Engineering:
  This research underscores the efficacy of a multistage feature extraction process that integrates AEs and LSTM networks. AEs encapsulate static attributes within the data, while LSTMs identify temporal patterns critical for detecting sequential anomalous activities. This dual-stage approach ensures a comprehensive representation of the dataset’s intrinsic characteristics. Additionally, the study addresses the issue of data imbalance by employing the Synthetic Minority Oversampling Technique (SMOTE) [24], which synthesizes samples for under-represented classes. SMOTE facilitates a balanced learning environment, thereby improving model performance on skewed datasets.
2. Enhanced Intrusion Detection:
  This study contributes to deep-learning-based intrusion detection by performing detailed class-specific calculations and analyses, a depth of analysis not commonly found in previous research. This rigorous approach strengthens the system’s ability to detect and classify intrusions accurately.

The proposed model evolves into a resilient anomaly detection system capable of identifying deviations from typical data patterns by integrating static and dynamic feature dimensions. The extracted features are infused into CNN algorithms, enhancing the model’s classification capabilities. This combination enables the system to distinguish benign traffic from malicious network traffic with high precision and reliability.

To ensure clarity and accessibility, the paper is structured as follows. Section 1 introduces the scope, objectives of the study and a comprehensive literature review of related work. Section 2 elaborates on the proposed methodology and framework, detailing the role of each component. Section 3 presents the experimental results, demonstrating the efficacy of the approach. Finally, Section 4 concludes the paper by summarizing key findings and discussing their implications.

## 2. Methods

Machine learning has emerged as a transformative approach to addressing complex challenges in network security. This section describes the methodology used in this research, encompassing the selection of datasets, the application of machine learning and deep learning techniques, and the systematic development of an effective detection methodology. The subsections below provide an in-depth discussion of the dataset, tools, preprocessing steps, and model architectures utilized in this study.

### 2.1. Machine Learning Application Methods in Network Security

The integration of machine learning techniques into network security has transformed the detection and prevention of cyber threats, offering adaptable and scalable solutions for complex attack scenarios. In this section, we detail the resources and methodologies employed in this research. First, we discuss the CIC IoT-2023 dataset, a comprehensive resource that provides a realistic representation of IoT-based cyberattacks and serves as the cornerstone for training and evaluating machine learning models. We then outline the tools and frameworks used in anomaly detection, leveraging a combination of machine learning and deep learning approaches to enhance accuracy and reliability. By establishing a structured workflow, including dataset preprocessing and model training/testing, we aim to address key challenges, such as class imbalance, feature scaling, and the complexity of network traffic patterns.

i. CIC IoT-2023 Dataset

The purpose of the CIC IoT dataset 2023 [25] is to act as an authentic dataset and standard for enhancing the creation of security applications for analytics that can effectively handle extensive threats in IoT environments. The dataset includes 33 attacks categorized into 7 classes, encompassing DDoS, DoS, web-based, spoofing, brute force, recon, and Mirai attacks. All of these attacks were carried out by malicious IoT devices that were aimed at other IoT devices within a network consisting of 105 real IoT devices. Thus, the dataset constitutes a realistic IoT attack dataset using a diverse topology of real IoT devices, detailed documentation of 33 attacks, and data collection for evaluation.

The dataset’s creation, led by the Canadian Institute for Cybersecurity (CIC), leverages the institute’s established presence in cybersecurity and commitment to advancing IoT security research by providing this valuable dataset to support various initiatives and research endeavors in IoT security. We utilize a representative subset comprising 10% of the dataset for our research. Table 1 provides a comprehensive dataset description, delineating the data types and corresponding quantities and offering insight into the diverse data categories and their respective quantities within the dataset.

While the CIC IoT 2023 dataset is invaluable, it is important to acknowledge its inherent limitations and potential biases. The following are some potential biases and limitations of the CIC IoT 2023 dataset or other datasets:

▪ Realism and Scalability: While the dataset aims to provide a realistic environment by employing actual IoT devices in its topology, there are inherent challenges related to scalability and the simulated nature of attacks. One challenge is the static nature of the dataset, which may not fully capture the dynamic and evolving characteristics of real-world IoT networks. The dataset lacks real-world noise and variability, such as network jitter, packet loss, or unexpected environmental interactions, which can influence the behavior of IoT devices and network traffic. Additionally, the dataset may contain biases due to the overrepresentation of certain attack types, such as DDoS, compared to less frequent but equally critical attacks, like spoofing or reconnaissance. These factors could potentially impact the generalizability of the model, particularly when deployed in heterogeneous IoT environments. The effectiveness of machine learning models trained on this dataset depends heavily on how well these simulated attacks and network traffic patterns correspond to real-world scenarios. There might be differences in how IoT devices behave in the lab and in real-world environments.
▪ Bias and Representation: One of the primary concerns with any dataset, including the CIC IoT 2023 dataset, is the risk of bias, whether in the form of underrepresented attack types, overemphasis on certain IoT devices, or skewed traffic patterns. These biases can lead machine learning models to develop overfitted solutions that perform well on the dataset but poorly in practical applications.
▪ Data Quality and Labeling: The accuracy of the data labeling process also significantly impacts the quality of the training models. Any errors in attack categorization or data annotation can lead to misinterpretations by the learning algorithms, potentially compromising the effectiveness of the IDSs developed using this dataset.

ii. Anomaly Detection With Machine Learning And Deep Learning

Anomaly detection in network security often involves identifying patterns that deviate from normal behavior, a task well-suited to machine learning and deep learning models. The experimental environment used for training and testing is detailed in Table 2. Python 3.9.12 was used as the programming language, and Jupyter Notebook tools were used. We used the Pandas and Numpy frameworks and feature engineering to clean the data. The Matplotlib and Seaborn frameworks were used to visualize the data. For data analysis, we utilized the Scikit-Learn and Keras frameworks.

Python is supported by the open-source web application Jupyter Notebook, which also supports other programming languages [26]. Python is a free programming language with a simple syntax. Linux, Windows, and Mac OS are platforms where Python is available. Python is popular mainly because it already has many capabilities for today’s leading technologies, such as machine learning, robotics, and data science, whereas many old programming languages lag behind these technologies [27].

Working with relational or labeled data is easy and natural because of the basic, flexible, and expressive data structures provided by the Python module named Pandas. The aim was to use it as the basis for practical Python data analysis. It is also the most versatile and effective open-source tool for data analysis, and it is available in various programming languages [28]. Matplotlib is a widespread Python collection for building static, animated, and interactive visualizations [29].

Scikit-Learn is a popular machine learning library. Scikit-Learn offers machine learning algorithms, including classification, clustering, regression, and dimension reduction. Scikit-Learn also offers data preprocessing modules, feature extraction, hyperparameter optimization, and model evaluation [30].

The Google Brain Team published an open-source, end-to-end machine learning framework known as TensorFlow.2.6.0 Researchers can swiftly design and deploy machine-learning-powered apps in TensorFlow’s scalable library ecosystem and community resources [31].

Keras is a Python-based deep learning library on the TensorFlow framework. Keras was created to enable rapid testing from the concept to the conclusion required for effective research [32].

### 2.2. Detection Methodology

Building on the dataset and computational tools described above, this section details the detection methodology used in this research. The integration of AE, LSTM, and CNN models in this framework allows for a comprehensive approach for detecting anomalies, leveraging the strengths of each architecture.

The proposed AE-LSTM-CNN architecture is designed to leverage the unique strengths of each component to address the multifaceted challenges of intrusion detection in IoT environments. Autoencoders (AEs) serve as the foundational layer, excelling in unsupervised feature extraction by compressing high-dimensional input data into lower-dimensional representations while retaining critical characteristics. This preprocessing step reduces redundancy and enhances the computational efficiency of subsequent layers. The LSTM layer complements this by focusing on temporal dependencies within the data, capturing sequential patterns critical for identifying time-based anomalies often encountered in IoT networks. Finally, the CNN layer refines these extracted features, identifying intricate spatial relationships and hierarchical structures to improve classification accuracy. Together, this hybrid approach ensures a comprehensive analysis of network traffic, enabling the detection of diverse and complex cyberattack patterns.

The sequence of AE, LSTM, and CNN layers in the proposed framework is intentionally designed to align with the hierarchical and complementary nature of feature extraction required for intrusion detection in IoT environments. Autoencoders (AEs) are positioned first to perform unsupervised feature extraction, focusing on reducing data dimensionality while retaining essential static features. This preprocessing step is critical for noisy datasets like CIC IoT 2023, where high-dimensional data can obscure patterns relevant to classification. The less-dimensional data serve as a clean input for subsequent temporal modeling.

Long short-term memory (LSTM) networks are placed next to leverage their strength in capturing temporal dependencies within sequences. IoT networks often exhibit time-based patterns of malicious activity, such as bursts of anomalous traffic during a DDoS attack or gradual escalation in spoofing attempts. LSTMs are uniquely equipped to identify these time-series dependencies, enriching the features extracted by the AEs with temporal insights.

Finally, convolutional neural networks (CNNs) are employed as the last layer to refine the combined static and temporal features. CNNs excel in identifying spatial patterns and hierarchical feature relationships, making them ideal for the final classification task. By processing data through progressively specialized layers, the proposed sequence ensures that static attributes are effectively reduced, temporal patterns are fully captured, and spatial features are accurately classified.

This structured progression from AEs to LSTMs to CNNs is motivated by the need to balance computational efficiency with analytical depth. Placing CNNs at the final stage allows the framework to focus on intricate spatial feature relationships once static and temporal features have been comprehensively processed, enhancing the precision and reliability of intrusion detection.

Each stage of the hybrid architecture is tailored to capture specific attributes of network traffic data. The autoencoder stage focuses on static features, such as packet-level characteristics or overall traffic volume, which are invariant over time. The LSTM layer captures dynamic features, such as changes in traffic patterns or burst behavior, that are indicative of ongoing attacks. The CNN stage then identifies and refines multi-dimensional interactions within the data, distinguishing between benign and malicious traffic with high precision. By integrating these three stages, the model achieves a holistic representation of the dataset, providing enhanced detection capabilities even for subtle and mixed attack patterns.

**Figure 1:**
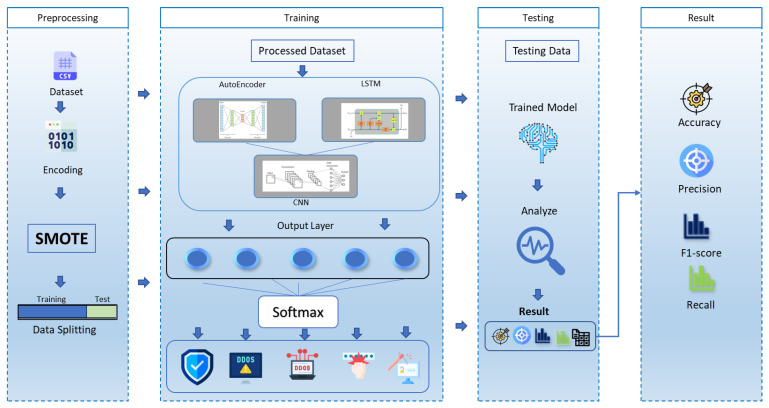
The proposed framework of hybrid AE-LSTM + CNN.

Single-model architectures, such as standalone CNNs or LSTMs, are often limited in their ability to address the diverse and dynamic nature of IoT cyberattacks. CNNs, while effective for spatial feature extraction, struggle to model sequential dependencies. Conversely, LSTMs excel in temporal analysis but may overlook finer spatial relationships. By combining these models with autoencoders, the hybrid architecture mitigates the weaknesses of individual models and creates a synergistic system capable of robust detection. This multistage approach also reduces overfitting, as each stage focuses on extracting complementary feature sets, ensuring generalizability across varied IoT environments. Figure 1 shows the proposed AE, LSTM, and CNN framework for preprocessing, training, and testing.

#### 2.2.1. Preprocessing

Effective preprocessing is a critical step in the machine learning pipeline, ensuring that the data are in a suitable format for analysis. This study employs a multistage preprocessing strategy, beginning with feature selection and target variable mapping to simplify the classification task. To address the issue of class imbalance in the dataset, SMOTE was applied, generating synthetic samples for underrepresented classes and creating a balanced dataset. The initial step involves defining the features (X_columns) and the target variable (y_column). The target variable is then mapped to seven broader classes of attack and one benign class, which helps reduce the problem’s complexity.

SMOTE is used on the dataset to address the challenge of class imbalance. SMOTE operates by creating synthetic instances in minority classes, thereby correcting imbalances in class distribution. While SMOTE effectively addresses class imbalance by synthesizing additional samples for minority classes, it is not without limitations. One potential drawback is the introduction of noise or unrealistic synthetic samples, which may not accurately represent real-world data distributions. This risk is particularly relevant in IoT environments, where attack patterns often exhibit complex temporal and spatial dependencies that SMOTE may fail to capture.

To mitigate these challenges, we carefully validated the synthetic data by examining the class distributions and ensuring that the generated samples align with the original data’s statistical characteristics. However, we acknowledge that the use of SMOTE might still impact the model’s performance when applied to highly dynamic real-world IoT scenarios. After SMOTE resampling, the features are standardized using the StandardScaler provided by the sklearn library. This standardization ensures that all features exhibit a mean of 0 and a standard deviation of 1. This normalization step is critical for certain machine learning algorithms that are sensitive to variations in the scale of input features.

The next step involves encoding the target variable using LabelEncoder. Next, one-hot coding is applied to convert the categorical labels into the appropriate format. Finally, the dataset is partitioned into training and testing sets, allocating 70% of the data for training purposes and setting aside the remaining 30% for testing.

This preprocessing step is an important part of the machine learning/deep learning workflow, ensuring that the data are in an appropriate format and sufficiently balanced.

#### 2.2.2. Training and Testing

**Figure 2:**
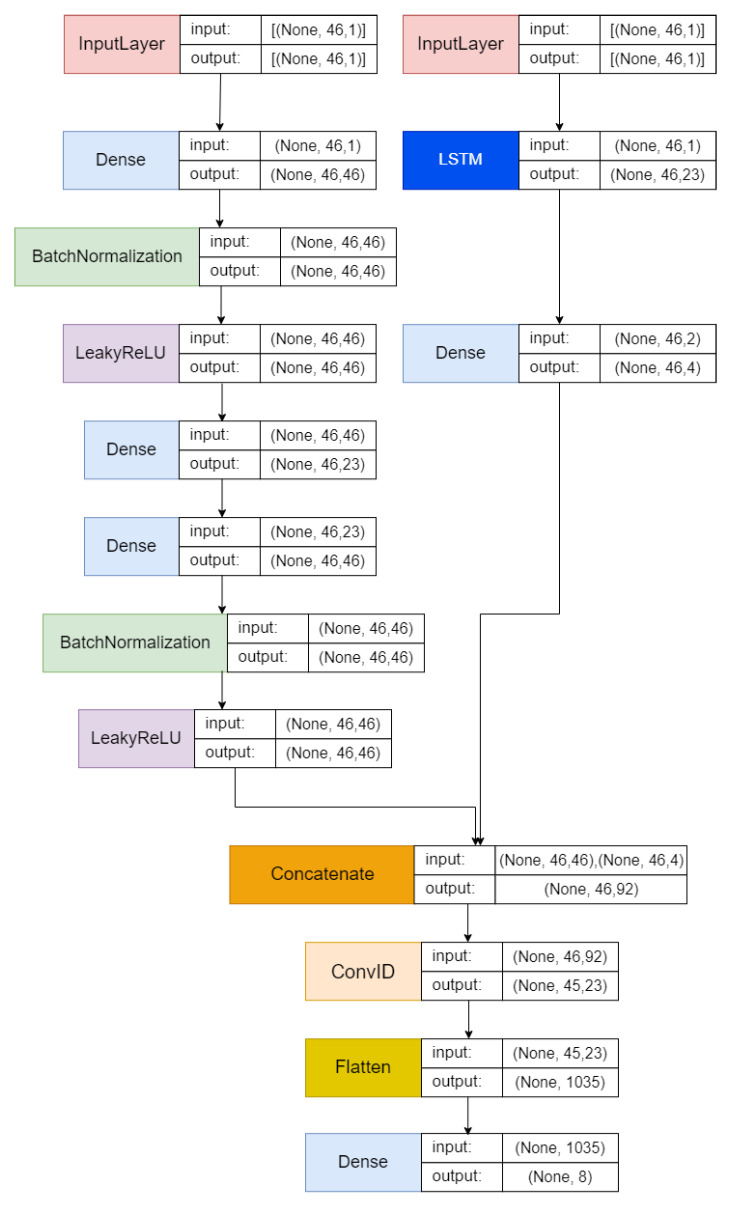
Proposed hybrid architecture AE-LSTM + CNN.

Figure 2 illustrates the proposed architecture integrating AE, LSTM, and CNN architectures. This combined architecture potentially presents a robust framework for enhancing performance in anomaly detection tasks. The fusion of these architectures aims to leverage the strengths of each model in addressing anomaly detection challenges.

The input layers are created according to the number of features in the input dataset. The architecture incorporates an AE, which is on the left side, consisting of an encoder, a bottleneck, and a decoder. The encoder applies a dense layer on the visible input, followed by batch normalization and the LeakyReLU activation function. The bottleneck determines its size as half of the input amount and applies a dense layer to the encoder output. Next, the decoder mirrors the encoder structure by utilizing dense layers on the bottleneck output, followed by batch normalization and the LeakyReLU activation function.

On the right side of the architecture, the input passes through the LSTM layer. This LSTM layer uses a hyperbolic tangent (tanh) activation function and produces a sequence as the output. Next, a dense layer is applied to the output generated by the LSTM.

The outputs from the decoder and LSTM layers are combined to create the main layer of the model. Next, a 1D convolutional layer utilizing a rectified linear unit (ReLU) activation function is applied to this combined output. The resulting output is then flattened.

The output layer consists of a dense layer that uses a softmax activation function adjusted to the number of output labels. The model was compiled using a stochastic gradient descent (SGD) optimizer, configured with a learning rate of 0.01 and a momentum of 0.9. The loss function chosen is “categorical_crossentropy”. Model performance is assessed using various metrics, including accuracy, the F1 score, recall, and precision, during evaluation.

#### 2.2.3. Rationale for Model Component Sequencing

The AE-LSTM-CNN framework is designed to leverage the strengths of each component in a parallel arrangement for effective feature extraction and classification. In this architecture, the autoencoder (AE) and long short-term memory (LSTM) layers operate simultaneously, each focusing on complementary aspects of the data.

Autoencoders are utilized for static feature extraction, compressing high-dimensional data into lower-dimensional representations while retaining the essential characteristics. This process reduces redundancy and noise, enabling more efficient processing in subsequent stages. Simultaneously, the LSTM layer processes the raw input to capture temporal dependencies, focusing on sequence-based patterns critical for detecting anomalies in IoT traffic. The parallel design ensures that both static and temporal features are extracted independently but concurrently, providing a holistic representation of the data.

The outputs from the AE and LSTM layers are concatenated to form a unified feature set, which is then passed to the CNN layer. The CNN refines these combined features by identifying intricate spatial relationships and hierarchical structures, enabling precise classification of IoT network traffic. This parallel integration of AE and LSTM ensures that the framework simultaneously exploits the strengths of both static and dynamic feature extraction methods, enhancing the robustness and accuracy of intrusion detection.

The parallel structure of AE and LSTM reflects the need to process both static and temporal attributes without introducing dependencies or bottlenecks. By combining their outputs before feeding them into the CNN, the framework ensures that the CNN layer receives a comprehensive and enriched feature set, maximizing its ability to differentiate between benign and malicious traffic.

For Algorithm 1, AE-LSTM + CNN shows the end-to-end architecture and relationships between layers. The output produced by the AE-LSTM + CNN algorithm is obtained through objective function modeling. Metrics, such as accuracy, the F1 score, recall, and precision, are used to measure the proposed architecture. The study is configured with a scenario using a predefined dataset. This measurement serves as an evaluative metric for assessing how effectively the model performs in carrying out its tasks together.
**Algorithm 1:** AE-LSTM + CNNInput: dataset Output: model and performance metrics1: 2: 3: 4: 5: 6:  7:
8:
9: 10: 11:
12:
13:
14:
15:
16:
17:
18:
19: 20:**# Determine the number of input features and output labels based on the dataset** n_inputs = X.shape^1^ n_output = y_hot.shape^1^ # Define the input layer for the neural network visible = Input(shape = (n_inputs, 1)) **# Define the encoder (left layer)** e = Dense(n_inputs)(visible) e = BatchNormalization()(e) e = LeakyReLU()(e) **# Calculate the bottleneck size** n_bottleneck = round(float(n_inputs)/2.0) **# Create the bottleneck layer** bottleneck = Dense(n_bottleneck)(e) **# Define the decoder** d = Dense(n_inputs)(bottleneck) d = BatchNormalization()(d) d = LeakyReLU()(d) **# Define the LSTM layer (right layer)** lstm = LSTM(round(n_bottleneck), activation = ‘tanh’, return_sequences = True, input_shape = (n_inputs, 1))(visible) lstm = Dense(n_inputs)(lstm) **# Concatenate the outputs from the AE and LSTM** concat = Concatenate()([d, lstm]) **# Apply a 1D convolutional layer** conv = Conv1D(filters = n_bottleneck, kernel_size = (2), activation = ‘relu’, input_shape = (n_inputs, n_inputs))(concat) conv = Flatten()(conv) **# Define the output layer** output = Dense(n_output, activation = ‘softmax’)(conv) **# Create the neural network model** model = Model(inputs = visible, outputs = output) **# Define the optimizer and compile the model** opt = SGD(learning_rate = 0.01, momentum = 0.9) model.compile(optimizer = opt, loss = ‘categorical_crossentropy’, metrics = [‘acc’, f1_m, recall_m, precision_m]) 

Accuracy refers to the degree of agreement or closeness between the predicted outcomes and the actual values in the dataset. This is related to the classification model. The formula for evaluating accuracy is shown in the following equation:

(1) $$\mathrm{Accuracy}=\frac{TP+TN}{TP+TN+FP+FN}$$

Precision represents the proportion of accurately identified positive cases relative to the total number of cases that were predicted to be positive. It is an essential metric in the evaluation of models. Precision was calculated using the following equation:

(2) $$\mathrm{Precision}=\frac{TP}{TP+FP}$$

Recall is a quantitative measure that evaluates a model’s capacity to accurately detect all pertinent instances of the target class inside of a given dataset. A higher recall score signifies that the model possesses the ability to accurately detect a greater percentage of true positive samples within the dataset. This shows better performance in recognizing the target class. It was calculated using the following formula:

(3) $$\mathrm{Recall}=\frac{TP}{TP+FN}$$

The F1 score is a performance metric that calculates both recall and precision when determining the accuracy of a model. A greater F1 score indicates enhanced overall efficacy in accurately classifying positive samples, rendering it a valuable metric for assessing the model’s capability to detect the target class. It was calculated using the harmonic mean of precision and recall and is expressed by the following formula:

(4) $$F1-\mathrm{score}=2*\frac{Precision*Recall}{Precision+Recall}$$

## 3. Results

Our integration model of autoencoders and LSTM and CNN architectures enables a robust detection mechanism that significantly improves accuracy, precision, recall, and F1 scores compared to traditional methods. The results demonstrate that the proposed AE-LSTM + CNN framework achieves remarkable performance across key metrics, including accuracy, precision, recall, and the F1 score. These improvements are attributed to the complementary roles of each model in the hybrid architecture. For example, the LSTM effectively recognizes temporal attack signatures, while the CNN refines spatial patterns in the input data. The experimental results are summarized in Table 3, highlighting significant advancements over benchmark methods. These findings underscore the practical significance of the proposed framework in real-world IoT environments. By outperforming state-of-the-art methods on the CIC-IoT 2023 dataset, the model demonstrates its potential for broader applicability. However, further research is necessary to explore its scalability and deployment in diverse network settings, particularly in environments with limited computational resources. This is particularly crucial given the escalating complexity and sophistication of cyber threats in IoT domains, where traditional security solutions often fail to detect and mitigate attacks effectively. By leveraging deep learning techniques, our approach not only enhances detection capabilities but also contributes to the ongoing development of adaptive security measures that are capable of handling emerging threats dynamically.

Table 3 presents the comparative performance of the proposed AE-LSTM-CNN framework against several state-of-the-art methodologies. The reported metrics of accuracy, precision, recall, and the F1 score were calculated using the testing subset of the CIC IoT 2023 dataset, representing 30% of the total data. Each metric reflects the framework’s ability to classify benign and malicious traffic accurately. Before model evaluation, the dataset underwent comprehensive preprocessing, including resampling using SMOTE, normalization, and encoding, as described in Section 2.2.1. These steps were crucial to ensure a balanced and representative testing set that accurately reflects the model’s generalization capabilities.

Table 3 compares the performance of our proposed AE-LSTM + CNN framework with state-of-the-art methodologies across key metrics: accuracy, precision, recall, and the F1 score. The following are detailed breakdowns and analyses of the results to highlight the specific advantages of the proposed method.

1. Accuracy Analysis

Observations:

▪ The proposed AE-LSTM + CNN achieves an accuracy of 99.15%, outperforming other methods, such as IoT-IDCS-CNN (98.20%) and DNN-BiLSTM (93.31%).
▪ The high accuracy of our framework is attributed to the hybrid architecture, which effectively integrates autoencoders (AEs), LSTMs, and CNNs. By leveraging AEs for robust feature extraction, LSTMs for temporal analysis, and CNNs for hierarchical classification, our approach captures both static and dynamic characteristics of IoT traffic.

Significance:

▪ Accuracy is critical in IoT environments, where misclassifications can lead to undetected attacks or unnecessary false alarms. A 99.15% accuracy ensures reliable performance across diverse attack types, significantly enhancing the system’s reliability in real-world scenarios.

Comparison:

▪ Methods like CNN-RNN and AE + ANN (accuracy: 98.22%) show strong performance but fall short in handling imbalanced datasets and complex temporal patterns. By incorporating SMOTE and LSTM layers, our framework addresses these limitations.

2. Precision Analysis

Observations:

▪ The proposed method achieves a precision of 99.39%, surpassing AE + ANN (97.50%) and CNN-RNN (98.00%).
▪ High precision reflects the model’s ability to minimize false positives (FPs). This is particularly important in IoT intrusion detection, where false positives can overwhelm security systems and erode trust in detection mechanisms.

Case Study:

▪ For attacks like Mirai and DDoS, the model demonstrates exceptionally low FP rates. For example, the Mirai attack class achieves a precision of 99.98%, with only 17 false positives out of thousands of predictions.

Comparison:

▪ While DNN-BiLSTM achieves strong recall, its precision (91.80%) is lower due to higher false positive rates. This highlights the advantage of our framework’s multistage architecture in refining feature extraction and classification.

3. Recall Analysis

Observations:

▪ Our framework achieves a recall of 99.00%, which is significantly higher than DNN-BiLSTM (93.05%) and IoT-IDCS-CNN.
▪ High recall indicates the model’s effectiveness in correctly identifying attack instances, minimizing false negatives (FNs). This ensures that the system detects most threats, even in complex IoT environments with imbalanced data.

Detailed Performance:

▪ For major attack types like Mirai and DoS, recall exceeds 99%. This demonstrates the model’s robustness in detecting time-sensitive and sequence-based anomalies.

Comparison:

▪ Traditional methods often struggle with recall due to their inability to handle imbalanced datasets or model sequential data effectively. By integrating SMOTE and LSTM layers, our framework addresses these issues, achieving superior recall performance.

4. F1 Score Analysis

Observations:

▪ The F1 score of 99.19% reflects a balanced performance across precision and recall. This makes the model particularly suitable for environments where both false positives and false negatives have significant implications.
▪ For example, in detecting Mirai attacks, the F1 score of 99.98% showcases the model’s ability to maintain consistent performance across all metrics.

Comparison:

▪ Methods like AE + ANN (F1 score: 97.68%) and DNN-BiLSTM (F1 score: 91.73%) show imbalances between precision and recall, leading to lower overall performance. Our hybrid approach ensures that both aspects are optimized simultaneously.

5. Advantages of the Proposed Methodology

Comprehensive Feature Integration:

▪ The AE-LSTM + CNN framework uniquely combines the strengths of static (AE), temporal (LSTM), and spatial (CNN) analysis. This integration enables the model to outperform others in handling diverse IoT attack types.

Handling Data Imbalance:

▪ The application of SMOTE ensures fair representation of underrepresented attack classes, as evidenced by improved metrics for classes like recon and spoofing attacks.

**Figure 3:**
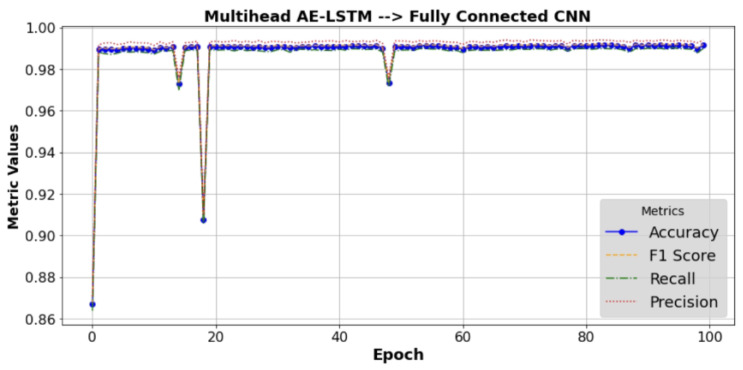
Performance result of the proposed model AE-LSTM + CNN.

The performance outcomes of the hybrid model over time are represented in Figure 3, with the F1 score and metric values (accuracy, precision, and recall) on the *Y*-axis, respectively. The illustration demonstrates the convergence and stability of the model throughout multiple training iterations or epochs. It indicates how these performance metrics evolve or stabilize as the model undergoes training.

From the evaluation results for the accuracy, F1 score, recall, and precision of several models, it can be observed that the AE-LSTM + CNN hybrid model exhibits higher accuracy than other models. The performance study compares the proposed AE-LSTM + CNN model with existing benchmark algorithms, such as CNN-RNN and AE-ANN. Based on these results, the AE-LSTM + CNN model outperforms these benchmark frameworks in terms of performance metrics. The results of a comparison of the performance of other researchers are presented in Table 3. This table showcases the performance of our proposed AE-LSTM + CNN model against various state-of-the-art methodologies in network intrusion detection using different source datasets. As illustrated, our model achieves an accuracy of 99.15%, precision of 99.39%, recall of 99.00%, and an F1 score of 99.19% with respect to the CICIoT2023 dataset. These results surpass the performance metrics of other recent approaches, such as that of Al-Haija et al. [11] with IoT-IDCS-CNN, Gamage et al. [21] with AE + ANN, Wang et al. [22] with DNN-BiLSTM, and Khan et al. [23], particularly with respect to precision and recall. When compared to other models, such as that of Wang et al. [22] using DNN-BiLSTM and that of Khan et al. [23] using DNN, both of which also utilize the CICIoT2023 dataset, our model demonstrates an improvement. These comparisons underline our model’s enhanced capability to detect and classify complex intrusion patterns more accurately, offering improvements, particularly in precision and recall, which are key indicators of model reliability in security-sensitive environments. We utilized a 10-fold method to evaluate performance and ensure objective outcomes. Our proposed model demonstrated good performance, as shown in Table 4.

To validate the robustness of the performance improvements of the proposed AE-LSTM + CNN model over Wang et al. [22] using DNN-BiLSTM, we performed statistical significance testing using paired t-tests and calculated 95% confidence intervals. The statistical tests were conducted across 10-fold simulated results for accuracy, precision, recall, and the F1 score. Table 5 summarizes the statistical significance analysis that shows statistical significance (*p* < 0.05) for accuracy, precision, recall, and the F1 score.

In addition to evaluating the AE-LSTM-CNN framework’s detection performance, we quantified its computational demands to provide a comprehensive assessment of its feasibility for deployment in resource-constrained IoT environments. The model required an average training time of 150 s per epoch on a system equipped with an Intel i9 CPU and an NVIDIA RTX 3060 GPU with 6 GB of memory. In comparison, the baseline DNN-BiLSTM model [22] exhibited higher training times. These results demonstrate that the proposed framework balances high detection accuracy with acceptable computational efficiency, making it a viable solution for IoT intrusion detection.

Our AE-LSTM + CNN model’s higher precision and recall indicate an enhanced capability in correctly identifying and classifying network threats with fewer false positives and negatives. This represents a significant improvement in ensuring reliable network security in IoT environments, which is critical given the increasing complexity and volume of network traffic. By integrating AE-LSTM with CNN, our approach effectively captures both temporal and spatial features in network data, which is a crucial enhancement over existing models that rely solely on one type of data representation.

**Figure 4:**
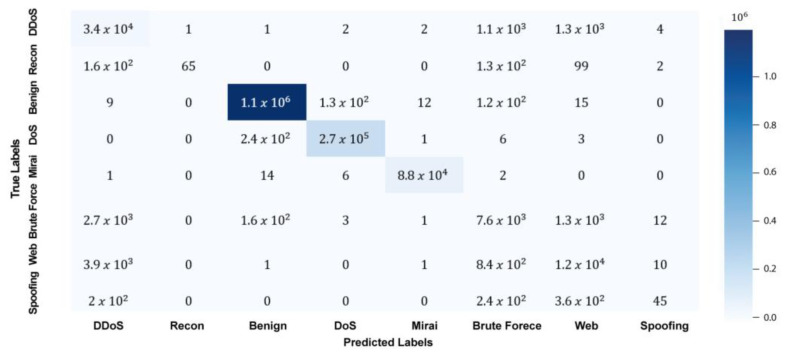
Confusion matrix of the AE-LSTM + CNN results.

A confusion matrix is a table that displays the predicted and actual classification of a dataset. The confusion matrix is usually structured as a table with actual classes represented on one axis and predicted classes on the other axis. Each cell in the matrix represents the number of instances classified according to their corresponding row and column intersections. In this manner, the matrix can provide a detailed view of how well an algorithm performs and which classes are most prone to errors. Figure 4 displays the confusion matrix of the proposed AE-LSTM + CNN results.

Utilizing the data presented in Figure 4 and the output from the experiment, we derive the true positive (TP), true negative (TN), false positive (FP), and false negative (FN) metrics for individual classes, as detailed in Table 6. The following is a summary of how to calculate true positives (TPs), false positives (FPs), false negatives (FNs), and true negatives (TNs) from a confusion matrix, as stated in Section 3 of the Results.

1. True Positive (TP): The number of instances correctly predicted for a specific class. Found on the diagonal of the confusion matrix for that class (row = column).
2. False Positive (FP): The number of instances incorrectly predicted as the target class but belonging to other classes. Calculated by summing the column for the target class (excluding the diagonal).
3. False Negative (FN): The number of instances belonging to the target class but predicted as other classes. Calculated by summing the row for the target class (excluding the diagonal).
4. True Negative (TN): The number of instances correctly predicted as not belonging to the target class. Calculated as the total number of instances minus the sum of TP, FP, and FN for the target class.

These steps are repeated for each class to compute their respective TP, FP, FN, and TN values.

Based on the resulting TP, FN, FP, and TN metrics for each class, we computed the accuracy, F1 score, recall, and precision, detailed in Table 7. The average performance of the model across all metrics (accuracy, precision, recall, and the F1 score) in detecting benign, DoS, and Mirai attacks exceeds 99%. In contrast, the accuracy for DDoS, recon, brute force, web, and spoofing attacks exceeds 99%, whereas precision, recall, and the F1 score differ significantly. The conducted analysis indicates that the dataset’s size remarkably impacts the performance metrics of each class of attack. When we carry out detailed examinations, the number of samples varies, which greatly affects the accuracy, precision, recall, and F1 score metrics across different attack classes. Thus, these results highlight the significance of considering dataset sizes in order to enhance the effectiveness of deep learning models in cybersecurity.

The performance analysis across different attack types reveals notable variations in precision, recall, and F1 scores. For example, while the framework achieves high precision and recall for Mirai and DDoS attacks, performance metrics for spoofing attacks are comparatively lower. This discrepancy may be attributed to the inherent complexity and subtlety of spoofing attack patterns, which can closely resemble benign traffic and are often characterized by lower frequency in the dataset. Additionally, the imbalanced distribution of attack samples in the CIC IoT 2023 dataset, as indicated by the relatively smaller number of spoofing instances compared to DDoS or Mirai attacks, may have impacted the model’s ability to generalize effectively for these minority classes.

To address this, the proposed AE-LSTM-CNN model incorporates SMOTE to alleviate class imbalance. However, the effectiveness of SMOTE in generating realistic synthetic samples for spoofing attacks may be limited, as it primarily relies on linear interpolation of existing data points, potentially failing to capture the nuanced characteristics of these attacks. This highlights the need for alternative approaches, such as augmenting the dataset with adversarial examples or employing advanced generative techniques, to enhance the model’s robustness against underrepresented attack types.

## 4. Conclusions and Future Works

This research article centered its investigation on the critical issue of intrusion detection within the context of the IoT. The proliferation of interconnected IoT devices exposes them to myriad cyber threats. This study advocates for the integration of a deep-learning-based mechanism within the IoT environment to counter these threats.

The proposed approach, AE-LSTM + CNN, was specifically designed to identify various security threats. Given the increasing complexity of cyber threats in the IoT field, the fusion of deep learning techniques shows significant potential in strengthening security in this domain.

In evaluating the AE-LSTM + CNN approach, we conducted a performance assessment that focused on important aspects of intrusion detection. This evaluation includes key metrics: accuracy, the F1 score, recall, and precision. Accuracy measures a model’s proficiency in correctly categorizing normal and dangerous activities. The F1 score, which provides a balance between precision and recall, indicates a model’s ability to identify threats while minimizing false alarms. Recall evaluates the model’s accuracy in recognizing all threats, while precision assesses its ability to avoid misclassifying non-threats. These four indicators offer a thorough assessment of the model’s effectiveness in enhancing IoT networks as a defense against cyber threats.

With the increasing use of IoT devices, the proactive identification and mitigation of threats are increasingly necessary. The results obtained in this study underscore the potential of deep learning in improving security protocols in IoT environments. This research provides input into ongoing efforts to fortify IoT systems against evolving cyber threats by evaluating the proposed approaches using established performance metrics. The incorporation of such state-of-the-art techniques not only reinforces IoT network security but also emphasizes the necessity of staying ahead of cyber adversaries in an increasingly interconnected world.

The CIC IoT 2023 dataset provides a robust foundation for evaluating model performance, but real-world IoT systems often exhibit diverse attack patterns that are not fully represented in static datasets. To ensure broader applicability, future research could involve training the model with augmented datasets generated through techniques like generative adversarial networks (GANs) and synthetic traffic simulations. These approaches will help the model adapt to novel threats and varying network topologies, thereby improving generalization and reducing reliance on specific dataset characteristics.

The rapidly evolving nature of cyber threats necessitates models that can adapt over time. Continuous learning techniques, such as online learning and incremental updates, would enable the AE-LSTM-CNN framework to stay effective in dynamic environments. Transfer learning and federated learning approaches could also facilitate knowledge sharing across different IoT systems without compromising data privacy. These strategies would ensure that the model remains robust against emerging attack vectors while maintaining compatibility with existing deployment constraints.

Future work could involve pilot deployments of the AE-LSTM-CNN model in real-world IoT setups, such as smart healthcare and industrial IoT environments. These deployments will provide insights into the model’s operational reliability, latency, and computational requirements. Key performance indicators, such as detection accuracy, false positive rates, and processing speed, could be evaluated in live network conditions. This real-world testing phase will validate the model’s scalability and adaptability, ensuring its readiness for deployment in security-critical applications.

The use of SMOTE for handling class imbalance in the CIC IoT 2023 dataset has proven effective in improving the detection of minority attack types. However, the introduction of synthetic samples may inadvertently introduce bias or unrealistic patterns that do not fully represent real-world attack behaviors. This limitation could affect the generalizability of the model, particularly in live IoT environments with rapidly evolving threats. To address this, future research could focus on integrating more sophisticated data augmentation methods, such as GANs, to generate synthetic samples that incorporate temporal and spatial dependencies. Additionally, alternative imbalance-handling strategies, such as cost-sensitive learning or ensemble-based techniques, could be investigated to further enhance model robustness.

Future research in this field may explore the adaptability and robustness of deep-learning-based IDSs across diverse IoT environments, accounting for variations in network scales and data types. The findings from this study highlight the critical role of hybrid deep learning frameworks in advancing IoT security. By addressing limitations in existing IDS methodologies, the proposed framework demonstrates its capacity to adapt to real-world operational constraints while maintaining superior detection performance. As IoT networks continue to expand and evolve, this research is a stepping stone toward developing more sophisticated and universally applicable IDS solutions. There is potential for further research to focus on developing dynamic learning models that continuously adapt to counter emerging attack strategies, thereby ensuring the sustainability and effectiveness of security measures in the ever-evolving IoT landscape. Additionally, exploring the integration of anomaly detection techniques or employing transfer learning methodologies could yield valuable insights into enhancing the generalization and scalability of these systems across varied IoT deployment scenarios.

The adaptability of the AE-LSTM-CNN framework could be further enhanced through the incorporation of transfer learning techniques. Transfer learning would allow the framework to leverage pre-trained models on similar IoT datasets, reducing the need for extensive retraining and accelerating the deployment process in new environments. For example, pre-trained models designed for general IoT intrusion detection tasks could be fine-tuned to detect specific attack types or operate in specialized settings, such as industrial IoT or healthcare systems. This approach could also mitigate the challenges associated with limited data availability in emerging IoT domains.

To improve the robustness of the model against adversarial attacks, adversarial training could be integrated into the learning pipeline. Adversarial training involves exposing the model to perturbed versions of input data during training, helping it learn to classify even in the presence of adversarial noise. This technique is particularly important in IoT environments, where attackers may attempt to manipulate data streams to evade detection. By incorporating adversarial training, the AE-LSTM-CNN framework could enhance its resilience and maintain high performance in adversarial scenarios.

Given the dynamic nature of IoT environments, continual learning could play a vital role in maintaining the framework’s efficacy over time. Continual learning allows the model to adapt to new attack patterns and network conditions without forgetting previously learned information. Techniques like elastic weight consolidation and incremental learning could be employed to enable the AE-LSTM-CNN framework to evolve in real time, ensuring its relevance in rapidly changing IoT ecosystems. This capability would be particularly valuable for long-term deployment, where static models often degrade in performance due to shifts in attack characteristics.

Building on the adaptive thresholding approach by Xu et al. in [33], future research could explore integrating interval-based observer techniques into intrusion detection systems. This would enable models to dynamically adjust detection criteria based on the evolving nature of network traffic and attack patterns, enhancing diagnostic accuracy and reducing the risk of misclassification in real-time applications.

Wang et al. [34] introduced a hybrid deep learning model combining ResNeSt and BiGRU with CTGAN for data balancing. Building on this, future research could explore similar hybrid frameworks and advanced techniques to enhance the detection of complex IoT attack patterns. Leveraging adaptive learning and feature extraction methods could address evolving threats, imbalanced datasets, and resource constraints in IoT environments.

## Figures and Tables

**Table 1:** Dataset description.

Type of Data	Value
Benign	111,208 data
Brute force attack	1285 data
DDoS attack	3,439,685 data
DoS attack	817,568 data
Mirai attack	266,306 data
Recon attack	35,855 data
Spoofing attack	49,444 data
Web attack	2471 data
Total	4,723,822 data

**Table 2:** Evaluation environment’s description.

Parameters or Variables	Value
**Hardware Specification**	
a. CPU model name	Intel(R) i9-11900H CPU @2.50 GHz (8 CPU core)
b. RAM	40 gigabytes
c. GPU	Nvidia RTX 3060
d. GPU memory	6 GB
**Model Evaluation Parameters**	
a. Epoch	100
b. Train–validation ratio	7:3
c. Optimizer	ReLu and Adam
d. Loss evaluation metric	Sparse categorical cross-entropy

**Table 3:** Comparison of proposed models.

Methodology	Algorithm	Source Data	Acc (%)	Pr (%)	Rc (%)	F1 (%)
Al-Haija et al. [11]	IoT-IDCS-CNN	NSL-KDD	98.20	-	-	-
Hiromoto et al. [12]	GAN	KDD	99.22	-	-	-
Pektas et al. [18]	CNN-RNN	CTU-143 and ISOT	99.00	98.00	97.00	97.00
Gamage et al. [21]	AE + ANN	CIC-IDS2018	98.22	97.50	98.22	97.68
Wang et al. [22]	DNN-BiLSTM	CICIoT2023	93.31	91.80	93.05	91.73
Khan et al. [23]	DNN	CICIoT2023	83.07	83.62	83.07	83.00
Proposed	AE-LSTM + CNN	CICIoT2023	99.15	99.39	99.00	99.19

Acc: accuracy; Pr: precision; Rc: recall; F1: F1 score.

**Table 4:** Proposed 10-fold models.

Fold	Accuracy (%)	F1 Score (%)	Recall (%)	Precision (%)
1	99.15%	99.18%	98.99%	99.39%
2	99.14%	99.18%	98.97%	99.39%
3	99.16%	99.20%	99.01%	99.40%
4	99.17%	99.20%	99.00%	99.40%
5	99.18%	99.21%	99.02%	99.41%
6	99.16%	99.19%	98.98%	99.41%
7	99.15%	99.19%	98.99%	99.39%
8	99.15%	99.19%	99.00%	99.39%
9	99.16%	99.19%	99.00%	99.39%
10	99.16%	99.19%	99.00%	99.39%

**Table 5:** Statistical analysis of the proposed model vs. Wang et al. [22].

Metric	Mean Difference (%)	Standard Deviation (%)	95% CI Lower (%)	95% CI Upper (%)	T-Statistic	*p*-Value
Accuracy	5.862	0.046	5.828	5.895	400.43	1.92 × 10^−20^
Precision	7.564	0.046	7.531	7.597	518.37	1.88 × 10^−21^
Recall	5.946	0.044	5.915	5.978	424.33	1.14 × 10^−20^
F1 Score	7.456	0.075	7.403	7.510	314.80	1.68 × 10^−19^

**Table 6:** Calculation of the confusion matrix.

Class	TP	FN	FP	TN
DDoS attack	34,150	2469	7040	1,515,203
Recon attack	65	387	1	1,558,409
Benign	1,135,136	286	420	423,020
DoS attack	268,951	251	141	1,289,519
Mirai attack	88,136	23	17	1,470,686
Brute force attack	7618	4249	2462	1,544,533
Web attack	11,513	4784	3139	1,539,426
Spoofing attack	45	799	28	1,557,990

**Table 7:** Performance results for each class.

Class	Acc (%)	Pr (%)	Rc (%)	F1 (%)
DDoS attack	99.39%	82.91%	93.26%	87.78%
Recon attack	99.98%	98.48%	14.38%	25.10%
Benign	99.95%	99.96%	99.97%	99.97%
DoS attack	99.97%	99.95%	99.91%	99.93%
Mirai attack	99.99%	99.98%	99.97%	99.98%
Brute force attack	99.57%	75.58%	64.19%	69.42%
Web attack	99.49%	78.58%	70.64%	74.40%
Spoofing attack	99.95%	61.64%	5.33%	9.81%

## Data Availability

Data were obtained from the Canadian Institute for Cybersecurity (CIC), University of New Brunswick, and are available at https://www.unb.ca/cic/datasets/iotdataset-2023.html (accessed on 6 January 2024).
